# Research retention among people with first-episode psychosis in KwaZulu-Natal, South Africa

**DOI:** 10.4102/sajpsychiatry.v31i0.2470

**Published:** 2025-08-29

**Authors:** Mihoko Maru, Vuyokazi Ntlantsana, Usha Chhagan, Lindokuhle Thela, Enver Karim, Andrew Tomita, Saeeda Paruk, Bonginkosi Chiliza

**Affiliations:** 1 Center for Innovation in Social Work and Health, School of Social Work, Boston University, Boston, Massachusetts, United States of America; 2 Department of Social Work, Thompson School of Social Work & Public Health University of Hawai’i at Mānoa, Honolulu, Hawai’i, United States of America; 3 Discipline of Psychiatry, Nelson R Mandela School of Medicine, University of KwaZulu-Natal, Durban, South Africa; 4 KwaZulu-Natal Research Innovation and Sequencing Platform, College of Health Sciences, University of KwaZulu-Natal, Durban, South Africa; 5 Centre for Rural Health, School of Nursing and Public Health, University of KwaZulu-Natal, Durban, South Africa

## Introduction

Research retention is a major methodological challenge in mental health research. High rates of study discontinuation or drop-out, contribute to significant missing data, impacting study validity and rigour. Retention is especially difficult in community-based longitudinal studies involving populations with complex medical conditions or those underserved in healthcare. Among participants with psychotic disorders in high-income countries (HICs), factors affecting retention include symptom-related issues such as paranoia, low motivation and limited ability to understand the study purpose.^1^ Study-related factors, such as the length of study participation and having multiple assessments, further make it challenging for researchers to retain participants, particularly in mobile populations (e.g., homeless population, refugees). Common socio-demographic characteristics associated with low retention in health research include, lower education, unemployment and being unmarried.^2,3,4^

In low- and-middle-income countries (LMICs), resources to promote participant study retention are limited. Researchers often rely on social workers and community health workers, who are already overburdened because of the disproportionately small healthcare workforce, for retention efforts. Much of what is known about barriers to retention in these low-resource contexts – such as inaccurate contact information, participant relocation, poor telecommunication infrastructure and the lack of reliable transportation^5^ – derives from longitudinal studies involving individuals living with human immunodeficiency virus (HIV). However, the issue of sustaining study engagement in mental health research has received little attention in LMICs. More data are needed to improve retention strategies to minimise participant disengagement in mental health research in LMICs.

This study sought to better understand the trends and patterns seen in research participation among a clinical sample of patients with first-episode psychosis (FEP) in KwaZulu-Natal, South Africa. We explored the rates of study retention in a sample of South African adults with FEP and the socio-demographic and clinical characteristics that may be associated with study retention.

## Method

The present study utilised data from a large longitudinal cohort study (*The burden of HIV and Psychosis in an African setting: A longitudinal study of HIV infected and non-infected patients with First Episode Psychosis* or *HIV in FEP study*) with male and female adults (ages 18–45 years) presenting with FEP and receiving psychiatric outpatient or inpatient care at five regional hospitals in KwaZulu-Natal, South Africa. A detailed methodology of this study providing information on study sites, participant inclusion and exclusion criteria and measures is described in an earlier publication by Chhagan et al.^6^ Data collection began in March 2019 and was suspended between April 2020 and October 2020 because of the coronavirus disease 2019 (COVID-19) pandemic and lockdown. For this study, we extracted and created a subset of the ‘HIV in FEP study’ data by selecting participants who enrolled from October 2020, after the COVID-19 restrictions were lifted, to December 2022. The total sample size for this subset was *N* = 97. Only socio-demographic data, including age, sex, marital status, educational background, current employment status, residential area, antipsychotic medication history, among others, were included in analysis (see Table 1). Data were collected in-person by clinicians through interviews at the hospitals, at baseline (within 6 weeks of clinical presentation), and at 3-months, 6-months and 12-months post-baseline. Participants were reimbursed R300.00 per visit for time, travel and inconvenience. Written informed consent was obtained from all participants prior to data collection. In cases where participants had guardians, verbal consent was obtained from the guardians to ensure they could be contacted if the participant forgot to follow-up.

**TABLE 1:** Baseline socio-demographic and clinical characteristics.

Variable	Mean	SD	*n*	%
Age	25.8	6.45	-	-
**Sex**				
Male	-	-	71	73.2
Female	-	-	26	26.8
**Race**				
Black people	-	-	91	93.8
Mixed race people	-	-	4	4.1
Indian people	-	-	2	2.1
**First language**				
IsiZulu	-	-	76	78.4
English	-	-	17	17.5
Other	-	-	4	4.1
**Education**				
Completed minimum grade school (7 years schooling)	-	-	82	84.5
Completed tertiary education	-	-	15	15.5
Attended tertiary education	-	-	31	32.0
**Employment**				
Unemployed	-	-	62	63.9
Student	-	-	15	15.5
Employed/Self-employed	-	-	20	20.6
Receives disability grant (No)	-	-	96	99.0
**Household income per month (South African Rands)**				
< R1500	-	-	11	12.2
R1501–R3000	-	-	18	20.0
R3001–R5000	-	-	16	17.8
R5001 <	-	-	45	50.0
**Marital status**				
Single	-	-	85	87.6
Married	-	-	6	6.2
Divorced	-	-	1	1.0
Cohabiting	-	-	3	3.1
Other	-	-	2	2.1
**Residential area**				
Urban	-	-	80	82.5
Rural	-	-	17	17.5
**Referral pathway**				
Family	-	-	70	72.2
Self	-	-	4	4.1
Other	-	-	23	23.7
Consulted with a traditional healer regarding symptoms (Yes)	-	-	41	42.3
History of childhood trauma (Yes)	-	-	63	65.6
Living with HIV	-	-	17	17.5
**Antipsychotic medication†**				
Typical oral antipsychotic	-	-	15	16.0
Atypical oral antipsychotic	-	-	85	88.5
Typical long-acting injectable antipsychotic	-	-	4	4.1

HIV, human immunodeficiency virus; SD, standard deviation.

† , Categories are not mutually exclusive.

### Analysis

Descriptive statistics were generated using frequencies to examine sample characteristics and trends of study participation post-baseline. A count variable for completed follow-up assessments was created for each participant, that is, 0 for those who only completed baseline, 1 for those who completed baseline and the 3-month follow-up, among others. Number of completed follow-up assessments were then divided by the total sample size (*N* = 97) to calculate retention rates. We ran bivariate analyses between all socio-demographic variables and the completed follow-up assessments variable using Spearman’s correlation and chi-square tests. We then ran negative binomial regression models with socio-demographic variables that were significantly associated with the completed follow-up assessments variable. Negative binomial regression is used for modelling over-dispersed count outcome variables and provides the ratio of the rate at which the outcome events occur, also known as the incidence rate ratio. Statistical significance was determined to be met at α = 0.05 level. All analyses were conducted in using SAS/STAT software, Version 9.4. Copyright © 2025 SAS Institute Inc.^7^

### Ethical considerations

Ethical clearance for this study was granted by the University of KwaZulu-Natal Biomedical Research Ethics Committee (reference number BCA 517/18) and the five clinical sites.

## Results

### Sample characteristics

The sample consisted of 97 participants, majority being black males (93.8%) with a mean age of 25.8 (standard deviation [SD] = 6.45) years. Majority of the sample lived in an urban area (82.5%) and were single (87.6%). The highest level of education completed was grade school for 84.5% of the sample, and while 32.0% reported they attended tertiary education, only 15.5% reported completing it. Nearly two-thirds of the sample were unemployed, and half reported an estimated household income of less than R5000.00 per month. A history of childhood trauma was also reported by two-thirds of the sample. At baseline, 88.0% were prescribed atypical antipsychotic medication (See Table 1).

### Rates of study retention

**FIGURE 1:**
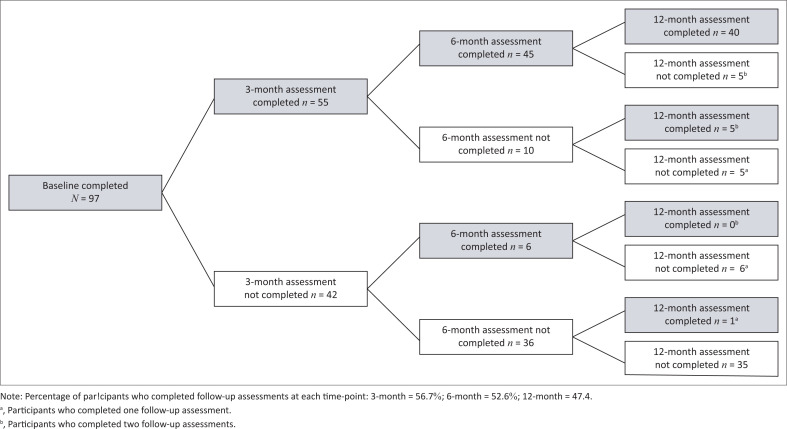
Study retention over the 12-month study period.

Figure 1 presents the trend seen in study retention. When observed cross-sectionally by assessment time point, 56.7% (*n* = 55) of the total sample completed the 3-month assessment, 52.6% (*n* = 51) completed the 6-month assessment, and 47.4% (*n* = 46) completed the 12-month assessment. Counting by number of assessments completed by individual, 41.2% (*n* = 40) completed all four assessments (baseline, 3-months, 6-months, 12-months), 12.3% (*n* = 12) completed baseline and one follow-up, and 10.3% (*n* = 10) completed baseline and two follow-ups. A little over a third (36.1%, *n* = 35) of the participants completed none of the follow-up assessments post-baseline.

### Socio-demographic correlates of completed follow-up assessments

Bivariate analyses showed that only two variables showed significant association with participants’ number of completed follow-up assessments: Having attended tertiary education and taking long-acting injectable (LAI) antipsychotic medication at baseline. Of those who attended tertiary education (*n* = 31), only *n* = 7 completed no follow-ups, while *n* = 3 completed one follow-up, *n* = 1 completed two follow-ups and *n* = 20 completed all three follow-up assessments post-baseline (Fisher’s exact *p* = 0.015, Cramer’s V = 0.33). These results suggested that those who attended tertiary education were more likely to complete follow-up assessments for the study than those who did not attend tertiary education.

At baseline, only four participants reported taking depot antipsychotic medication. Because of the low sample size, results for the association between LAI antipsychotic medication and the count of completed follow-up assessments were not interpreted.

Negative binomial regression with attendance of tertiary education regressed on completed follow-up assessments showed that the incident rate for those who attended tertiary education was 1.59 times the incident rate for those who did not attend tertiary education. Over the study period, those who attended tertiary education had 59.0% greater incidents of follow-up assessments completed compared to those who did not attend tertiary education. Results also indicated that the outcome variable was over-dispersed (dispersion coefficient > 0) and that the application of negative binomial regression was appropriate (dispersion coefficient alpha = 0.08, 95% confidence interval [CI] = 0.003, 2.862), and good model fit (*p* = 0.4645).

## Discussion and conclusion

We analysed trends of study retention using longitudinal data from 97 adults with FEP receiving psychiatric care in KwaZulu-Natal, South Africa. Overall, our findings highlight significant challenges with participation retention. Over the 12-month period, approximately 40.0% of the sample completed all four assessments, while about 25.0% completed only one or two follow-ups. By the first follow-up, 43.3% were lost to follow-up, and one-third were entirely lost to after baseline assessment. These retention rates are comparable to those reported in studies from HICs, where dropout among individuals living with psychosis ranges from 36.0% – 68.0%.^1^ We also found that higher educational attainment was associated with better retention, consistent with previous research.^1^ Among individuals living with serious mental illness, higher education has been associated with enhanced cognitive functioning and greater self-efficacy,^8,9^ both of which may support sustained engagement in longitudinal research.

This study has several limitations. Participants were only followed up at baseline, 3, 6, and 12 months, and not monthly, which may have affected study retention rates. Furthermore, as the study sites were located in urban areas, some participants may have had to travel long distances for follow-up visits, contributing to loss to follow-up. It is also important to note that the data reflect retention in the study, not necessarily retention in treatment. Our study focused on participants who completed a follow-up assessment within the study, and did not formally capture whether they remained engaged in treatment. It is possible that some participants, who were temporarily residing in the district for work, subsequently returned to their homes in other districts or provinces and continued receiving care there, but were unable to complete their study follow-up visit.

Effective strategies to maximise participant retention in longitudinal cohort studies have centred on reducing barriers for participants. These strategies include subsidising transportation costs, offering childcare services or using alternative methods for data collection (e.g., phone interviews^10^). Emerging strategies such as social media, text messaging and study websites have also shown promise in improving retention.^10^ Low-resourced research settings, such as South Africa could benefit from a more robust infrastructure to track patients through a centralised healthcare system database. In addition, flexible and low-cost retention strategies, such as involving traditional healers or community support groups – who are playing an increasingly important role in mental healthcare – could further enhance participant retention.^11^ Our findings were similar to trends observed in treatment disengagement when psychiatric patients transition from inpatient to outpatient services. Currently, the only retention strategy employed in our study setting is appointment reminders via text messages and/or phone calls. Additional creative and feasible strategies are needed to retain individuals immediately following baseline and/or post-discharge to improve both research and clinical outcomes. Further research is essential to understand the factors contributing to study retention in mental health research and services in LMICs, as well as to explore participants’ perspectives on effective strategies that facilitate better engagement in both research and treatment.
